# Antimicrobial Use and Antimicrobial Resistance in Food-Producing Animals: Cross-Sectional Study on Knowledge, Attitudes, and Practices Among Veterinarians and Operators of Establishments in the Republic of Cyprus

**DOI:** 10.3390/antibiotics14030251

**Published:** 2025-03-01

**Authors:** Despina Theodoridou Oxinou, Demetris Lamnisos, Charalampos Filippou, Nikolaos Spernovasilis, Constantinos Tsioutis

**Affiliations:** 1 Veterinary Services, Nicosia 1417, Cyprus; 2 Public Health Program, European University Cyprus, Nicosia 2404, Cyprus; d.lamnisos@euc.ac.cy; 3 School of Sciences, European University Cyprus, Nicosia 2404, Cyprus; 4 School of Medicine, European University Cyprus, Nicosia 2404, Cyprus; c.filippou@euc.ac.cy; 5 Department of Infectious Diseases, German Oncology Center, Limassol 4108, Cyprus; nikolaos.spernovasilis@goc.com.cy

**Keywords:** antimicrobial resistance, antibiotic use, surveillance, food-producing animals, veterinarians, operators, one health

## Abstract

**Background**: Current evidence suggests that more than half of all antimicrobials are used in the sector of food-producing animals, thus constituting a major risk factor for development and spread of antimicrobial resistance (AMR). **Methods**: This cross-sectional study assessed the knowledge, attitudes, and practices regarding antimicrobial resistance (AMR) and antimicrobial use (AMU) among veterinarians (n = 26) working with food-producing animals and operators (n = 165) of establishments that keep food-producing animals, across all districts of Cyprus between October and December 2024. **Results**: Most veterinarians demonstrated sufficient knowledge regarding AMR and AMU; however, certain knowledge gaps were identified. There was a general trend toward desired responses aimed at reducing AMU. Despite this, the level of responses advocating for restrictions on the use of specific priority antimicrobials and broad-spectrum antibacterials was unsatisfactory. Over half of veterinarians prescribed Category B substances. Furthermore, there was no significant association between the use of “restricted” antibiotics and veterinarians’ level of knowledge nor between antibiotic use and the practice of microbiological culture and susceptibility testing. Among operators, positive attitudes were significant predictors of implementing of good practices. Additionally, operators who had contracts with a veterinarians were more likely to follow good practices. **Conclusions**: There is a need for further education on AMR in the veterinary sector in the Republic of Cyprus. Raising awareness among animal producers, is also crucial, along with enforcing a policy on antimicrobial use. Comprehensive governance involving all stakeholders must be implemented to address AMR more effectively.

## 1. Introduction

Antimicrobial resistance (AMR) is recognized globally as one of the most serious public health challenges. In 2019, AMR was identified by the World Health Organization (WHO) as one of the most serious global threats to public health, and in July 2022, it was identified by the European Commission as one of the three major threats to public health in the European Union (EU) [1]. AMR poses a cross-border threat to public health, animal health, plant health, and the environment. Infections due to resistant bacteria account for 700,000 deaths globally every year and could cumulate to 10 million by 2050 if no constant efforts to tackle AMR are implemented [2,3]. The estimates from the European Union/European Economic Area (EU/EEA) indicate that every year, more than 670,000 infections are due to resistant bacteria, and approximately 33,000 people die as a direct consequence. [4]. The health burden of antimicrobial resistance is caused by 40% of infections with bacteria resistant to critically important antibiotics [5]. According to the Organization for Economic Co-operation and Development (OECD), the rates of AMR infections will continue to rise in the EU/EEA, with a significant impact on the budget of healthcare systems [5]. Without immediate effective action, the annual cost of AMR is expected to increase to EUR 1.1 billion between 2015 and 2050 in all EU and EEC countries [5].

The four main sectors involved in the spread of AMR are the human medicine sector in the community and in the hospital settings, animal production, agriculture, and the environment [6]. According to The State of the World’s Antibiotics 2015, two thirds of all antibiotics produced worldwide each year (65,000 tons out of 100,000 tons) are used in livestock farming [7]. Similarly, the global AMU in bovine animals, poultry, and porcine animals was estimated at 93,309 tons in 2017, and another study showed that the same animal groups, along with ovine animals, consumed 99,502 tons of antibiotics in 2020 and was predicted, based on those trends, to increase to 107,472 tons by 2030 [8]. It was also estimated that worldwide, the antimicrobial use in food-producing animals exceeds the antimicrobial use in human health [9]. The sixth report of WOAH, which includes the AMU reported by 109 participant countries for 2018, showed that 69,455 tons of antimicrobials were used in food animals in 2018. WOAH estimates that the adjusted total amount could be 76,704 tons [10]. According to the thirteenth ESVAC report which presents data on the sales of veterinary antibiotic agents from 31 European countries in 2022, overall aggregated sales of antibiotic veterinary medicinal products were 73.9 mg/PCU.

The transmission of antibiotic-resistant bacteria and genes from animals to humans has been well documented in the literature [11,12,13]. The transmission of AMR to establishments keeping food-producing animals has been studied across various species including [14,15] pigs, cattle [16], and arthropods [17,18,19]. The extent of AMR transmission between animals and humans is a topic of great interest, with significant implications for both public and animal health [20,21,22]. Notably, EFSA’s 2021 scientific opinion on the role of the environment in the emergence and spread of antimicrobial resistance through the food chain provides insight into the role of environmental factors in the spread of AMR in plant-based food production in the EU, terrestrial animals (poultry, cattle, and pigs), and aquaculture [23].

The development and spread of antimicrobial resistance is extensively seen by organizations like the European Food Safety Authority, the World Health Organization, and the Lancet Infectious Diseases Commission as a consequence of the use and overuse of antibiotics in both human and veterinary medicine. Scientific research led to a consensus among many scientists that, for certain bacterial infections, such as *Campylobacter* spp. and *Salmonella* spp., the use of antibiotics in animal farms is the primary cause of resistance to human infections. The emergence of resistance to critically important antibiotics in *Escherichia coli* and *Salmonella* spp. is a significant issue, resulting from the inappropriate use of these antibiotics in both human and veterinary medicine. Methicillin-resistant *Staphylococcus aureus* (MRSA) strains that affect humans are also becoming an increasing concern, due to the widespread use of certain antibiotics in food-producing animals [24]. The prudent use of antimicrobials along with high standards of infection prevention and control in both animals and humans, are crucial components in tackling the issue of antimicrobial resistance. Understanding the knowledge, attitudes, and practices (KAP) of veterinarians as well as those of operators of establishments keeping food-producing animals is crucial for developing effective interventions. [25]. Insufficient knowledge and awareness of antimicrobial use (AMU) and AMR have been reported as contributing factors to the inappropriate prescribing and dispensing of antibiotics. [26,27]. These groups are therefore considered key stakeholders, central to the efforts required to mitigate the emerging threat of AMR globally.

According to the thirteenth ESVAC report, Cyprus has the highest sales at 254.7 mg/PCU. Although overall sales in 2022 decreased by 14.1% in comparison to 2021 (from 296.5 mg/PCU to 254.7 mg/PCU) they are still at a high level. In 2022, the proportion of veterinary antimicrobials belonging to AMEG categories B was at 3.3%. The overall sales of the antibiotic classes of EMA AMEG Category B, i.e., third- and fourth-generation cephalosporins, fluoroquinolones, other quinolones and polymyxins among 31 countries, from <0.01 to 0.47 mg/PCU, <0.01 to 12.6 mg/PCU, 0 to 0.75 mg/PCU, and 0 to 10.2 mg/PCU, accounting for 0.17%, 2.8%, 0.16%, and 2.8% of total aggregated sales, respectively [28]. This report highlights certain classes of antimicrobials included in Category B of the categorization made by the EMA’s Antimicrobial Advice ad hoc Expert Group (AMEG) in 2019 [29]. The World Health Organization (WHO) categorization of antimicrobials (6th revision) [30] is taken into consideration, as well as the necessity for the respective antimicrobials in veterinary medicine and the probability of transferring antimicrobial resistance from animals to humans. Category B of the AMEG categorization includes those veterinary antibiotics from which the risk to public health is estimated to be higher than from other classes of antibiotics; fluoroquinolones, other quinolones, third- and fourth-generation cephalosporins, and polymyxins are included in this category. Third- and fourth-generation cephalosporins, and fluoroquinolones are considered by the WHO as ‘highest-priority critically important antimicrobials’ (HPCIAs) in human medicine. These groups have also been categorized as Veterinary Critically Important Antimicrobial Agents in the WOAH list of antimicrobials of veterinary importance [31].

According to the most recent Joint Interagency Antimicrobial Consumption and Resistance Analysis (JIACRA VI report) published in 2024, in 2021, the total antimicrobial consumption (AMC) was assessed at 125.0 mg/kg of biomass for humans (28 EU/EEA countries, range 44.3–160.1). In Cyprus, the total AMC was assessed at 139.9 of biomass for humans and 296.5 mg/kg of biomass for food-producing animals. The total AMC was assessed at 92.6 mg/kg of biomass for food-producing animals (29 EU/EEA countries, range 2.5–296.5). In 2021, the EU/EEA population-weighted mean consumption of fluoroquinolones and other quinolones was 6.3 mg/kg estimated biomass in humans and 2.9 mg/kg of estimated biomass in food-producing animals. The EU/EEA population-weighted mean consumption of fluoroquinolones and other quinolones was 6.3 mg/kg estimated biomass in humans and 2.9 mg/kg of estimated biomass in food-producing animals. The relevant mean for polymyxins was 0.7 mg/kg and 2.5 mg/kg, respectively. There was a statistically significant association between the consumption of fluoroquinolones in food-producing animals and fluoroquinolone resistance in *E. coli* from food-producing animals. The same results were observed for colistin for the time periods 2018–2019 and 2019–2020 [32].

Evidence recorded to describe antimicrobial resistance levels in the annual reports of the European Union on antimicrobial resistance in zoonotic and indicator bacteria from humans, animals, and food, shows very high resistance to fluoroquinolones (ciprofloxacin) among *Salmonella* isolates from broilers (50%) in Cyprus [32,33]. Multidrug resistance was observed at very to extremely high levels among *Salmonella* spp., *Campylobacter coli* and *Campylobacter jejuni*, and indicator *Escherichia coli* recovered from food-producing animals. Resistance to ciprofloxacin ranged from high to extremely high in *C. jejuni*, *C. coli* isolates, and *E. coli* commensal from food-producing animals. A statistically significant increase in resistance to ciprofloxacin was noted in *E. coli* isolates from broilers between 2014 and 2022. Additionally, resistance to erythromycin was observed at very high levels in *C. coli* from pigs.

To this end, evaluating the knowledge, attitudes, and practices (KAP) of veterinarians and operators of food-producing animal establishments is important for a better understanding the drivers of antimicrobial resistance in the animal health sector and providing evidence for the development of effective interventions.

## 2. Results

### 2.1. Socio-Demographic Characteristics of Respondents

Overall, all veterinarians (n = 26) working in the food-producing animal sector and 165 (approximately 50% of the total) operators of establishments keeping food-producing animals participated in the current study. The veterinarian’s socio-demographic characteristics are presented in Table 1.

The relevant characteristics of operators of establishments keeping food-producing animals, are presented in Table 2. The distribution of the studied animal population in each district is included in Table 3.

### 2.2. Knowledge, Attitudes, and Practices of Veterinarians on AMR and AMU

**Figure 1:**
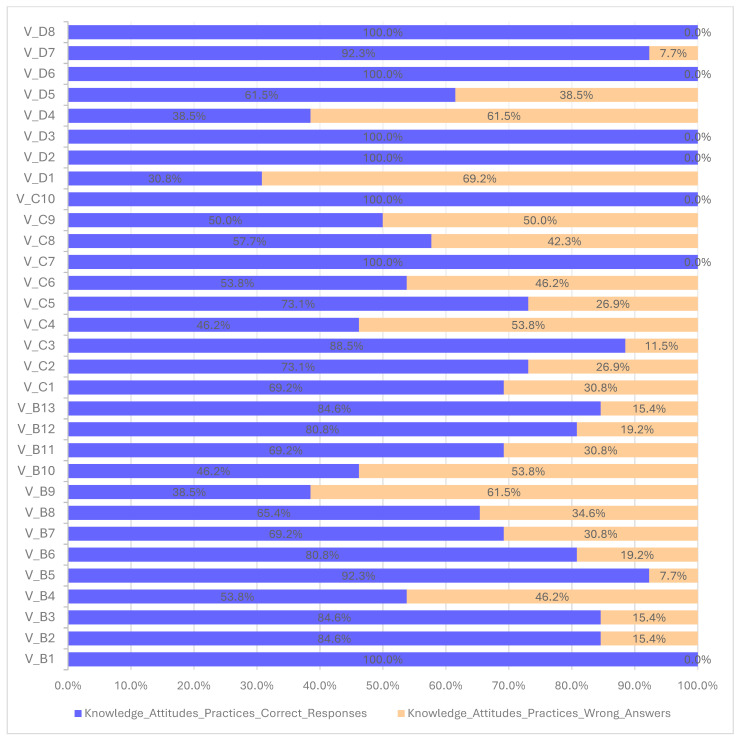
The proportion of veterinarians who correctly or incorrectly responded to knowledge, attitudes, and practices questions on AMR and AMU.

Generally, the submitted responses indicate a high level of knowledge (Table 4 and Figure 1), with more than 82% of answers being correct. The majority of veterinarians working with food-producing animals reported being aware of the severity of antimicrobial resistance.

Regarding the need to restrict the use of specific classes of antibiotics (HPCIAs; critically important for human medicine) as well as broad-spectrum antibiotics, there was a lack of positive attitudes, with a target response rate of only 57.7%. Additionally, only half of the participants agreed that critically important antibiotics should be restricted to human use only. Fewer than half of the participants responded negatively to the question of whether reducing the use of veterinary antibiotics by 50% would have negative effects on animal health and welfare (46.2%, n = 12). Similarly, the proportion of respondents who answered negatively to the question asking “if using two or more classes of antibiotics in combination is always better for infection control” was also low (53.8%, n = 14).

Interestingly, 61.5% of all respondents frequently prescribed more than one antimicrobial in a single prescription. Additionally, more than half of the veterinarians (57.7%, n = 15) prescribe Category B antibiotics (polymyxins, quinolones, fluoroquinolones), which are considered “restricted” according to the European Medicines Agency (EMA) categorization. On the other hand, 69.2% of veterinarians involved with food-producing animals prescribe Category D antibiotics (such as penicillins and tetracyclines), and 50% of them prescribe Category C antibiotics.

To initially investigate whether there is an association between the knowledge of the updated criteria for classifying antibiotics by the EMA and the use of “restricted” antibiotics (Class B), the chi-squared statistical test was applied. The results indicated no statistically significant association between the level of knowledge on antibiotic categorization criteria and the use of Class B antibiotics. This observation suggests that although there is knowledge, attitudes may influence practices that are not aligned with recommended guidelines.

The number of respondents using microbiological culture and susceptibility testing to select the appropriate antibiotics for treatment was low (30.8%, n = 8). When investigating the dependence between the use of microbiological culture and susceptibility testing and the use of Class B antibiotics, no statistically significant association was found between the relevant variables. This suggests that Class B antibiotics are often prescribed without prior susceptibility testing. As for Class D antibiotics (considered for “prudent use”), they are commonly used alongside Category B and Category C antibiotics, with only two veterinarians prescribing only Class D antibiotics.

### 2.3. Association Between Factors Related to Knowledge, Attitudes, and Practices

Table 5 presents the cross-tabulation of the use of “restricted” antibiotics in food-producing animals and various variables related to KAP. No statistically significant association was observed between the tested variables.

### 2.4. Knowledge, Attitudes, and Practices of Operators of Establishment Keeping Food-Producing Animals on AMR and AMU

**Figure 2:**
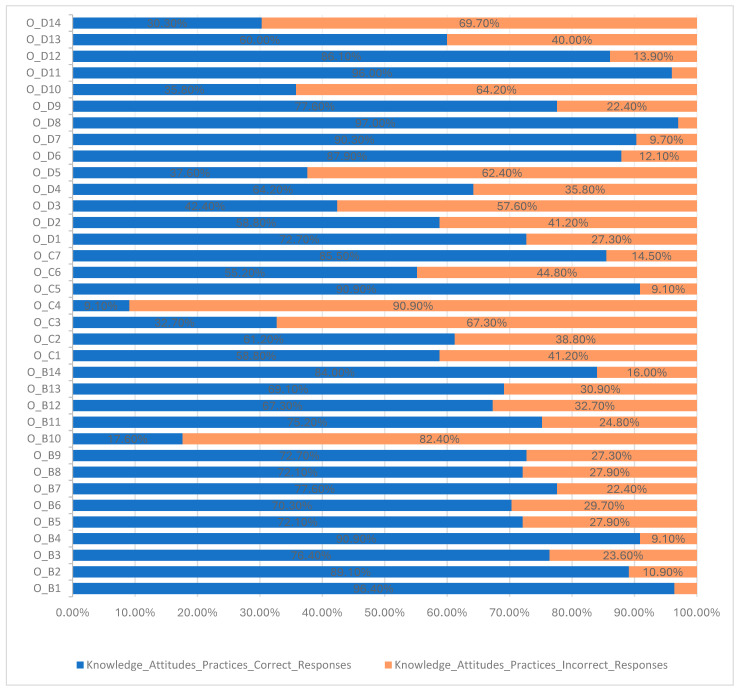
Proportion of operators of establishments keeping food-producing animals who correctly or incorrectly responded to knowledge, attitudes, and practices questions on AMR and AMU.

Based on responses, participants’ knowledge of AMR and the use of antimicrobials is generally moderate (Table 6 and Figure 2). It was also observed that a high percentage of operators (82.4%, n = 136) reported that antimicrobials are effective in treating both bacterial and viral infections, while 17.6% of all respondents gave the correct answer “No”.

The positive attitude corresponded to a correct response rate of more than 63%, while the negative attitude was associated with less than 63%. When asked whether participants would use fewer antimicrobials if they knew that accidental use could prevent recovery in the future, a high percentage responded positively (85.5%, n = 141). Additionally, 61.2% of all producers (n = 110) believed that “insufficient dosing can contribute to antibiotic resistance”, while 58.8% (n = 97) agreed that “randomly used antimicrobials could contribute to antimicrobial resistance”. A general trend was observed where most of the operators believed that antimicrobials should be stored in restricted areas and accessed only by specific staff when needed, though only 9.1% of respondents answered this correctly. Similarly, less than half of respondents (32.7%, n = 54) agreed with the statement that “limiting the use of antimicrobials can cause more harm than benefits”, indicating a negative attitude. This information is detailed in Table 6 and Figure 2.

Importantly, a significant proportion (72.7%, n = 120) of food-producing animal establishment operators reported having an agreement with a veterinarian to monitor their establishments. Additionally, 87.90% of respondents indicated that they did not receive recommendations on the antimicrobial from non-veterinarians, and almost all (96.4%, n = 159), stated that prescriptions are made according to the veterinarian’s instructions. However, 64.2% reported interruptions in antimicrobial treatment, while 22.4% of respondents mentioned increasing the dose and frequency of antimicrobials when there was no evidence of improvement in animal health. Furthermore, some operators (36.80%, n = 59) reported using antimicrobials in their animals on their own initiative. When asked if they reserved antimicrobials for later use, only 37.60% answered correctly.

Regarding the implementation of biosecurity measures, 60.00% of participants reported applying biosecurity measures in their establishments. However, a smaller proportion (30.30%, n = 50) demonstrated knowledge of what biosecurity measures entail, as indicated by their responses to the following question. The remaining 29.70%, of the 60.00%, either did not answer the question “mention biosecurity measures you apply to your holding” or provided incorrect answers.

### 2.5. Association Between Factors Related to Knowledge, Attitudes, and Practices for Operators of Establishments

According to the results of a logistic regression of adjusted odds ratios for certain categorical attitude variables in relation to the adoption of good practice on farms (Table 7), such as using alternatives to antibiotics, responders who answered the attitude questions correctly were 2.44 times more likely (OR = 2.44, 95% CI: 1.19, 5.00) and 2.33 times more likely (OR = 2.33; 95% CI: 1.12, 4.76) to implement good practices like using alternatives compared to those who did not answer the attitude questions correctly. Furthermore, operators who have an agreement with a veterinarian to monitor their establishments were 2.56 times more likely (OR = 2.56, 95% CI: 1.22, 5.56) to implement good practices such as using alternatives to antibiotics compared to operators who do not have a contract with a veterinarian for monitoring their establishments.

The logistic regression (Table 8) shows that positive attitude factors are important predictors for implementing good practices. Operators who answered the attitude questions correctly, as described in Table 8, are 2.33 times more likely (OR = 2.33, 95% CI: 1.03, 5.26) and 3.13 times more likely (OR = 3.13; 95% CI: 1.47, 6.67) to apply good practices such as implementing biosecurity measures compared to operators who did not respond correctly to the attitude questions.

Table 9 shows the cross-tabulation of the use of a “contract with a veterinarian who monitors the establishment” and several variables related to attitudes and practices. The data suggest that the likelihood of a negative attitude among operators without a contract with a veterinarian is 2.89 times greater (95% CI, 1.43–5.85) compared to those who have a contract with a veterinarian. Additionally, the likelihood that good practices are not adopted by operators without a contract with a veterinarian is 3.02 times greater (95% CI, 1.48–6.17) compared to those who have a contract with a veterinarian.

### 2.6. Association Between Socio-Demographic Characteristics of Operators of Food-Producing Animal Establishments with Knowledge, Attitudes, and Practices

The results in Table 10 show that the educational level of responders was positively associated with both their level of knowledge (*p* = 0.025), and attitudes (*p* = 0.032). However, no statistically significant association was found between the educational level and the level of practices. Regarding the animal species and farm districts, there was significant heterogeneity in the number of responders in each category. This variability may have impacted the results, as a statistically significant association was observed between the district and levels of attitudes, between the district and levels of knowledge, and between animal species and the levels of knowledge and attitudes. This could potentially lead to inaccuracies in generalizing the findings to the broader population.

## 3. Discussion

Antimicrobial resistance and the inappropriate use of antimicrobials are global challenges, and this issue is also evident in Cyprus [28]. Reducing AMR in both public and animal health necessitates a coordinated effort from all stakeholders, including operators of food-producing animal establishments and veterinarians. Gaining insights into the knowledge and awareness levels regarding antimicrobial resistance among veterinarians and individuals in rural areas is crucial. This information plays a vital role in the context of the One Health approach, which aims to address and reduce the growing threat of AMR.

This study is the first of its kind conducted in the Republic of Cyprus to assess the KAP of veterinarians and operators of food-producing animals establishments concerning AMR and AMU. The findings revealed that veterinarians generally exhibited a high level of knowledge, with most reporting an understanding of the severity of AMR. However, there was a noticeable gap in knowledge regarding the concept of antimicrobial stewardship. Antimicrobial stewardship is a critical component of a multisectoral strategy to combat AMR, involving a series of actions designed to promote the responsible and prudent use of antibiotics [34]. This highlights the need for further awareness and education on AMR and its management in veterinary practice [35].

All respondents agreed that improved husbandry practices and biosecurity measures can effectively reduce antibiotic consumption. This attitude was supported by a statistically significant positive association between the implementation of certain good practices by operators and the establishment of a contract with a veterinarian for monitoring purposes. Other studies have similarly shown that veterinarians have a substantial influence on the attitude of the livestock farmers [36,37,38,39], reinforcing the conclusion that continuous training of veterinarians on the severity of AMR and its connection to antimicrobial use is crucial. Furthermore, fostering communication between operators of food-producing animal establishments and veterinarians is vital for promoting responsible antimicrobial use [40].

Several veterinarians reported that they often prescribe more than one antimicrobial in a single prescription. Additionally, more than half of the veterinarians prescribe Category B antibiotics (polymyxins, quinolones, fluoroquinolones) which are classified as ‘restricted’ according to the European Medicines Agency (EMA) categorization [41]. These antibiotics are also considered as the highest-priority critically important antimicrobials (HPCIAs) by the World Health Organization (WHO) [42].

No statistically significant association was found between the knowledge of antibiotic categorization criteria. and the practices of antibiotic use. This suggests that despite existing knowledge, attitudes can still lead to unacceptable practices. Similar findings have highlighted the issue of prescribing critically important antimicrobials as the first line of treatment across Europe [43,44,45]. However, according to the latest 2022 report from EMA, between 2011 and 2022, sales of third- and fourth-generation cephalosporins decreased by 49.0%, sales of fluoroquinolones by 24.7%, sales of other quinolones by 89.7%, and sales of polymyxins by 81.0%. The inappropriate prescription of antibiotics by veterinarians could be a significant contributor to the development of antimicrobial resistance, as observed in previous studies [46,47].

The use of microbiological culture and susceptibility testing to select the appropriate antibiotics during treatment is not a common practice. A significant negative association was observed between the use of susceptibility testing and the use of Class B antibiotics. This suggests that Category B antibiotics are often prescribed without prior microbiological cultures and susceptibility testing. These findings contradict those of other researchers [48], who reported that the majority of veterinarians used the susceptibility testing to select appropriate antimicrobials. Sensitivity testing is an important tool in the veterinary field for selecting the most suitable antimicrobial product to treat bacterial diseases in animals. For Class D antibiotics (“prudent use”), they are often used alongside Category B and Category C antibiotics, while only 2 of the 26 veterinarians in our study used only Class D antibiotics. A study involving 25 European countries highlighted that the most critical factors influencing veterinarians’ selection of antibiotics in animal treatment are antibiotic susceptibility test results, their own experience, the risk of developing AMR, and easy administration [48]. This observation may suggest a need to improve access to services that provide susceptibility testing.

The responses gathered from operators of food-producing animal establishments suggest that participants’ knowledge on AMR and the use of antimicrobials is moderate. The majority of responsible farm operators of food-producing animals, based on their declarations, are aware of what antimicrobials are, the withdrawal times for antimicrobials, and the severity of antimicrobial resistance. However, according to other studies, although producers are aware that antibiotic misuse is linked to antibiotic resistance, they do not view it as a major problem. They are also not particularly concerned about the consequences of the reckless use of antimicrobial medicines and the impact of antimicrobial resistance on both animal and public health [49,50]. Less than half of the respondents believe that “accidentally used antimicrobials could contribute to antimicrobial resistance,” reflecting a lack of sufficient knowledge and negative attitudes toward the issue.

Approximately two-thirds of farm operators of food-producing animals have an agreement with a veterinarian who monitors their farm. Additionally, the majority of respondents reported not receiving recommendations for the use of antimicrobials from non-veterinarians, and almost all indicated that prescriptions are made according to the instructions of the veterinarian issuing them. However, there is evidence of the potential misuse of antimicrobial in the surveyed establishments, including practices such as maintaining doses of antimicrobial medicines for future use, interrupting the administration of antimicrobials, and increasing the dose and frequency of antimicrobials when there is no evidence of an improvement in animal health. Additionally, some operators reported using antibiotics without consulting a veterinarian. Parameters that could lead to such practices include inadequate knowledge on prudent use of antimicrobials, and the role of veterinarians in observing the establishments [40]. The results also show that the educational level of responders was positively linked to the level of knowledge, and the level of attitudes. The findings of several studies are consistent with this study’s results [27,51].

Good practices are among the most effective methods for preventing antibiotic misuse and overuse, thus helping to reduce AMR [52,53,54,55,56,57]. The current study emphasized the adoption of good practices by several participants, including the use of alternatives to antibiotics, which demonstrated a statistically significant positive association with attitude parameters. The study also highlighted that positive attitudes are key predictors for the implementation of good practices. Operators who answered attitude-related questions incorrectly were less likely to adopt good practices, such as the implementation biosecurity measures, compared to those who responded correctly. Factors that may influence the adoption of good practices, like biosecurity measures, include collaboration with veterinarians as a source of information, the operators’ experience, availability of time, and the cost of necessary investments. As observed in the current study, operators that have an agreement with a veterinarian to monitor their establishments are more likely to implement good practices such as the use of alternatives to antibiotics in relation to operators who do not have a contract with a veterinarian for monitoring their establishments. These findings agree with those of Rayner et al. (2019), who stated that operators who have regular twice-a-year or annual visits from their vet may have active, beneficial flock health plans in place including measures to prevent disease and reduce the use of antibiotics [58]. Regarding the use of probiotics, operators must be persuaded of the benefits of approaches that reduce antibiotic usage while ensuring safe and effective health outcomes through probiotic use. According to a relevant study on the benefits, costs, and considerations of using antibiotic alternatives in food-producing animals, further research is necessary to confirm that probiotics are viable long-term alternatives for operators in place of antibiotics [59].

### Limitations of the Study

One of the main limitations of this study is the response rate of operators in food-producing animals, which was 48%. This limits the ability to explore potential variations in characteristics across different participant categories. As a result, there may be a systematic non-response bias, as those who chose not to participate likely have different characteristics than those who did. However, compared to similar studies, this rate is considered relatively high [60,61]. Similarly, despite capturing the total number of veterinarians in the sector, their small sample size may lead to potential overestimation of findings related to their responses. Additionally there was heterogeneity in the number of participants from different districts and an unequal distribution of operators of establishments across different animal species. Since the sampling of the studied population was not random but rather based on convenience, this contributed to the uneven distribution across different data categories. This approach may have led to an overestimation of positive findings, as it likely included a higher proportion of individuals with a positive attitude toward antimicrobial resistance and antimicrobial use.

Misinterpretation and potential ambiguity of questions could also introduce systematic information bias. To minimize the risk, closed questions were used. However, closed questions may be prone to a systematic error related to social desirability bias, where participants may answer in the most socially acceptable way, especially given the sensitive nature of the prudent use of antimicrobials. This could result in responses that reflect what is perceived as the “correct” or socially approved answer rather that the participants’ true beliefs or practices.

## 4. Materials and Methods

### 4.1. Ethical Approval

This study was approved by the Cyprus National Bioethics Committee (CNBC 2024.01.299). Participant consent was obtained electronically from those who received the questionnaires via email, and via paper forms from those who were given printed questionnaires. The consent statement was included within the questionnaire itself.

### 4.2. Place and Period of the Study

The study was conducted across all districts of Republic of Cyprus (Nicosia, Limassol, Famagusta, Larnaca, and Paphos) between October and December 2024, using an online questionnaire. The study population consisted of operators of bovine, ovine, caprine, and porcine establishments, as well as operators of poultry establishments and registered veterinarians listed in the Veterinary Registry.

### 4.3. Sampling Method

For the selection of operators of food-producing animal establishments, a non-proportional stratified random sampling method (with respect to animal species) and proportional stratified random sampling method (with respect to different districts) were employed. Regarding the selection of registered veterinarians from the Cypriot Veterinary Register who are employed in the field of productive animals, data from the Pancyprian Veterinary Association indicated that there are approximately twenty-six (n = 26) such veterinarians and they constituted the target population of the study. Regarding operators, operators of bovine, caprine, and porcine animal establishments registered in the Animal Identification and Registration System of the Veterinary Services were eligible for inclusion in the study. Additionally, operators of poultry establishments who are registered in the central register maintained by the Veterinary Services were also eligible.

Due to reduced responses from the food-producing animal sector, convenience sampling was employed. The sample size for operators was determined to be 341 using the Raosoft electronic program (http://www.raosoft.com/samplesize.html) (accessed on 11 October 2024). This sample size was calculated based on a 50% response distribution, a 5% margin of error, and a 95% confidence interval. The 50% response rate was assumed because the actual response rate was unknown as there were no similar previously published studies from Cyprus to reference.

### 4.4. Questionnaire Development

Following a comprehensive literature review of comparable studies [49,62,63,64,65,66], two district questionnaires were developed: one for veterinarians working in the field of food-producing animals, and another for operators of food-producing animal establishments. The reliability of internal consistency was estimated using Cronbach’s alpha, calculated as follows:

$$a=\frac{k}{k-1}\left(1-\frac{\sum_{i=1}^{k}\sigma_{\iota }}{\sigma_{total}^{2}}\right)$$

where *k* is the number of questions in the scale, $\sigma_{\iota }^{2}$ represents the variation in each question, and $\sigma_{total}^{2}$, is the overall variation in the sum of all questions’ scores. Cronbach’s alpha was calculated to be 0.78 for knowledge, attitudes, and practices questions in the questionnaire for operators of food-producing animal establishments and at 0.69 for the questionnaire for veterinarians. The Cronbach’s alpha values of 0.69 and 0.78 are generally acceptable. The lower value in the range is slightly below the ideal threshold of 0.7.

The questionnaire includes a declaration of consent to participate in the study and is divided into four sections, predominantly consisting of closed-type questions. The first section addresses the socio-demographic characteristics of the participants. The second section focuses on gathering information related to knowledge of antimicrobial resistance and the use of antimicrobials. The third section is designed to assess respondents’ attitudes toward antimicrobial use and antimicrobial resistance. The fourth section examines the practices of animal producers concerning the topics being studied. Both negative and positive elements were included for each topic.

A score of “1” was assigned for each correct answer, while a score of “0” was given for incorrect or doubtful responses. Based on previous studies [67,68], a high level of knowledge was associated with a correct response rate above 82%, a moderate level with a correct response rate between 55 and 82%, and a low level of knowledge with a rate below 55%. Regarding good practices, the corresponding percentages were above 58%, between 35 and 58%, and below 35%. A positive attitude was defined by a correctness rate of more than 63%, while a negative attitude was associated with a correctness rate of less than 63%.

### 4.5. Method of Data Collection

Data were collected using Google Forms (retrieved 20 October 2024, from https://forms.google.com.

The link was sent via e-mail. For operators of animal establishments, printed questionnaires were also provided as an alternative method of data collection.

### 4.6. Statistical Analysis and Data Processing

The data were analyzed using the software GNU PSPP version 2.0.1-gff8d3d. The data collection involved two main categories of responses: ‘yes’, and ‘no’ or ‘don’t know’, concerning various knowledge, attitudes, and practices related to antimicrobial use and antimicrobial resistance. For the correct/positive or correct/negative responses, the value “1” was assigned, while for the incorrect/negative or incorrect/positive answers, or if the response was “don’t know”, the value “0” was given.

Initially, descriptive statistical methods were applied, using absolute frequencies (n) and relative frequencies (%) to evaluate demographic characteristics, as well as the level of knowledge, attitudes, and practices related to antimicrobial use and antimicrobial resistance.

To assess the influence of various factors of attitudes, knowledge, and practices on certain good practices (e.g., knowledge and implementation of biosecurity measures and the use of alternatives to antibiotics), a list of all potential independent variables was first compiled, and relevant tables of relevance (cross-tabulations) were created. The statistical test χ^2^ was applied with an alpha level of significance set at 0.05 for all inferential statistics. In the next step, multiple logistic regression models were created, including variables for which the probability of falsely rejecting the null hypothesis (i.e., the *p*-value) was less than α = 0.05.

Multivariable logistic regression was used, with responses related to attitudes as independent variables and a dichotomous assessment of the implementation of good practices as the dependent variable. The multivariable logistic regression models were used to estimate the adjusted odds ratio for the variables under examination, such as “agreement with a veterinarian”, and other attitude-related factors described with detail in Table 7 and Table 8. The results were expressed as odds ratios (ORs) with 95% confidence intervals (95% CIs), and a *p*-value of <0.05 was used as the threshold for statistical significance.

To explore the association between socio-demographic characteristics and knowledge, attitudes, and practices, the non-parametric Kruskal–Wallis test was employed.

## 5. Conclusions

It has emerged that the role of the veterinarian is crucial in encouraging producers to adopt good practices. Additionally, the role of veterinarians is essential in changing the behavior of stakeholders involved in veterinary practices. Changes in the attitudes and practices of veterinarians in Cyprus regarding antimicrobial use are essential. This can be achieved by further promoting continuous education and the dissemination of information within the veterinary sector about the use of antimicrobial medicinal products and AMR at both undergraduate and postgraduate levels. Additionally, promoting antimicrobial stewardship is crucial to educate and support veterinarians following adopting evidence-based practices for prescribing and administering the highest-priority critically important antimicrobials (HPCIAs). For operators of food-producing animal establishments, it is important to note that positive attitudes were predictors of good practices. However, further efforts are needed to strengthen collaboration between operators and veterinarians to achieve higher rates of good practices. Programs must be designed to raise awareness about the risks of antimicrobial resistance and the importance of responsible antibiotic use should be expanded. Operators of animal establishments should understand the broader impact of AMR on public health, animal health, and the environment.

Although improving the awareness and understanding of AMR is one of the primary objectives of the Cyprus AMR National Action Plan, the results of the current study suggest that there is still significant progress to be made. There is an urgent need to enhance the awareness and understanding of AMR through effective communication, education, and training, involving all stakeholders and facilitating behavioral change interventions. Previous research has shown that experience gained through appropriate training, access to published literature, and the availability of treatment guidelines play a crucial role in changing veterinarians’ prescribing behavior [69]. There is a need to establish evidence-based guidelines for prescribing antimicrobials, particularly for “restricted” and “highest-priority” antibiotics, which should emphasize the importance of microbiological cultures and susceptibility tests before prescribing antimicrobials. Veterinarians should be encouraged or required to use diagnostic tools. Financial or logistical support could be provided to make these tests more accessible to veterinary practices.

There is a strong need for policies that enforce the restricted use of the highest-priority antibiotics. These measures should be coupled with more robust governance to ensure that both veterinarians and farm operators adhere to best practices in antimicrobial use. Furthermore, continuous training and information dissemination should be prioritized in the veterinary sector to keep up with evolving guidelines and resistance patterns.

Governments, veterinary associations, and industry groups could launch communication campaigns targeting farm operators, focusing on the importance of following veterinarian advice and reducing antimicrobial use where possible. Veterinary associations, governments, and research institutions should work together to develop and implement policies, guidelines, and educational campaigns that promote the responsible use of antimicrobials in food-producing animals.

## Figures and Tables

**Table 1:** Socio-demographic characteristics of veterinarians participating in the study.

Variables		N (%)
Gender	Female	6 (23.1)
	Male	20 (76.9)
Age (years)	23–30	3 (11.5)
	31–45	14 (53.8)
	46–60	7 (26.9)
	61 or more	2 (7.7)
Educational level	Degree in veterinary medicine	17 (65.4)
	Master’s degree or doctorate	9 (34.6)
Area of residence	Rural	12 (46.2)
	Urban	14 (53.8)
Training on the use of antimicrobials		26 (100.0)
Percentage of veterinarians working at a veterinary pharmaceutical company		9 (34.6)

**Table 2:** Socio-demographic characteristics of operators participating in the study.

Variables		N (%)
Gender	Female	20 (12.1)
	Male	145 (87.9)
Age (years)	18–30	17 (10.3)
	31–45	52 (31.5)
	46–60	43 (26.1)
	61 or more	53 (32.1)
Educational level	Elementary school graduate	22 (13.3)
	High school graduate	85 (52.5)
	Undergraduate degree	47 (28.5)
	Master’s degree or doctorate	11 (6.7)
Area of residence	Rural	108 (65.5)
	Urban	57 (34.5)
District where establishments are located	Nicosia	64 (38.8)
	Larnaca	42 (25.5)
	Limassol	16 (9.7)
	Ammochostos	14 (8.5)
	Paphos	29 (17.6)
Types of establishments	Bovine animal establishments	32 (19.4)
	Ovine and caprine animal establishments	103 (62.4)
	Porcine animal establishments	16 (9.7)
	Poultry establishments	14 (8.5)
Professional experience (years)	<4	12 (7.3)
	5–8	15 (9.1)
	9–12	16 (9.7)
	>13	122 (73.9)
Main employment/occupation	Livestock farming	148 (89.7)
	Other	17 (10.3)

**Table 3:** Distribution of food-producing animal establishments which constituted the population studied.

	Types of Farms			
District	Number of Bovine Establishments n (%)	Number of Ovine and Caprine Establishments n (%)	Number of Porcine Establishments n (%)	Number of Poultry Establishments n (%)
Nicosia	14 (43.8)	32 (31.1)	8 (50.0)	10 (71.4)
Larnaca	9 (28.1)	25 (24.3)	7 (43.8)	1 (7.1)
Limassol	2 (6.3)	14 (13.6)	0 (0.0)	0 (0.0)
Ammochostos	2 (6.3)	10 (9.7)	0 (0.0)	2 (14.3)
Paphos	5 (15.6)	22 (21.4)	1 (6.3)	1 (7.0)
Total	32 (100.0)	103 (100.0)	16 (100.0)	14 (100.0)

**Table 4:** The proportion of veterinarians who correctly/positively responded to knowledge, attitudes, and practices questions on AMR and AMU.

**Knowledge Factors**		**N (%)**
V_B1	Do you know what antimicrobial resistance is?	26 (100.0)
V_B2	Are you aware of the World Health Organization’s (WHO) categorization of antimicrobials?	22 (84.6)
V_B3	Are you aware the categorization of veterinary antimicrobials by the World Organization for Animal Health (WOAH)?	22 (84.6)
V_B4	Are you aware of the updated criteria for the categorization of antibiotics used in veterinary medicine as established by an expert group (AMEG) of the European Medicines Agency (EMA)?	14 (53.8)
V_B5	Do you consider antimicrobial resistance to be a serious public health threat?	24 (92.3)
V_B6	Do you believe that the use of antibiotics in animals is a major contributor to the development of resistance to bacterial infections in humans?	21 (80.8)
V_B7	Are you aware of information about methicillin-resistant *Staphylococcus aureus* (MRSA)?	18 (69.2)
V_B8	Do you believe that the use of expired antimicrobials contributes to the development of antimicrobial resistance?	17 (65.4)
V_B9	Do you know what antimicrobial stewardship is?	10 (38.5)
V_B10	Are you familiar with the recommendations outlined in the Council Recommendation (2023) on strengthening EU action to combat antimicrobial resistance within the context of the One Health approach?	12 (46.2)
V_B11	Do you believe that antimicrobials are effective in treating both bacterial and viral infections?	18 (69.2)
V_B12	Do you believe there is an ongoing misuse of antibiotics in the veterinary sector?	21 (80.8)
V_B13	Do you think that antibiotic residues in food contribute to the development of antimicrobial resistance in humans?	22 (84.6)
**Attitude Factors**		**N (%)**
V_C1	The potential contribution of veterinary antimicrobial medicinal products to the development of resistance in humans is concerning.	18 (69.2)
V_C2	I support the policy of reducing veterinary antibiotic consumption by 50% by 2030, compared to 2019 levels.	19 (73.1)
V_C3	My goal is to minimize the use of antibiotics as much as possible within the context exercise of my profession.	23 (88.5)
V_C4	Reducing veterinary antibiotic use by 50% will lead to negative effects on animal health and welfare.	12 (46.2)
V_C5	I have become more aware of the need to impose restrictions to the use of antibiotics in recent years.	19 (73.1)
V_C6	Do you think that using two or more classes of antibiotics in combination is always more effective for infection control?	14 (53.8)
V_C7	Do you believe that a thorough examination of animals is necessary before prescribing or administrating an antibiotic?	26 (100.0)
V_C8	Do you think that broad-spectrum antibiotics are better than narrow-spectrum antibiotics, even when narrow spectrum drugs are available and suitable?	15 (57.7)
V_C9	Do you believe priority antibiotics should be restricted to human use only?	13 (50.0)
V_C10	Do you think that improved livestock practices and the implementation of biosecurity measures can help reduce antibiotic consumption?	26 (100.0)
**Practices Factors**		**N (%)**
V_D1	Do you use microbiological culture and susceptibility testing to select the appropriate antibiotics for treatment?	8 (30.8)
V_D2	Do you check the expiry date of antibiotics before using them?	26 (100.0)
V_D3	Do you advise farmers on the withdrawal period for antimicrobial medicines?	26 (1000)
V_D4	Do you provide advice to farmers on how to use antimicrobial medicines over the phone?	10 (38.5)
V_D5	Do you often prescribe more than one antimicrobial in a single prescription?	16 (61.5)
V_D6	Do you advise farmers to complete the full course of antimicrobials that you prescribe?	26 (100.0)
V_D7	Are you using alternatives to antimicrobials in your practice?	24 (92.3)
V_D8	Do you advise farmers on appropriate vaccination strategies to help reduce the use of antimicrobials?	26 (100.0)

**Table 5:** Cross-tabulations: the variable “use of class B antibiotics” and variables related to knowledge, attitudes, and practices among veterinarians.

		Use of Class B Antibiotics		*p*-Value	Odds Ratio for (No/Yes)	95% Confidence Interval	
		No	Yes			Lower Bound	Upper Bound
Veterinary Pharmaceutical companies	No	6 (75.0%)	11 (73.3%)	0.931	1.09	0.15	7.80
	Yes	2 (25.5%)	4 (26.7%)				
Knowledge—EMA classification	No	4 (50.0%)	6 (40.0%)	0.645	1.50	0.27	8.45
	Yes	4 (50.0%)	9 (60.0%)				
Knowledge—Antimicrobial stewardship	No	6 (75.0%)	10 (66.7%)	0.676	1.50	0.22	10.30
	Yes	2 (25.0%)	5 (33.3%)				
Knowledge—Council Recommendations (2023)	No	3 (37.5%)	10 (66.7%)	0.179	0.30	0.05	1.80
	Yes	5 (62.5%)	5 (33.3%)				
Attitude—Reducing by half the use of veterinary antibiotics	Yes	1 (12.5%)	4 (26.7%)	0.433	0.39	0.04	4.28
	No	7 (87.5%)	11 (73.3%)				
Attitude—Use of two or more classes of antibiotics in combination	Yes	4 (50.0%)	6 (40.0%)	0.645	1.50	0.27	8.45
	No	4 (50.0%)	9 (60.0%)				
Attitude—Broad-spectrum antibiotics vs. narrow spectrum	Yes	3 (37.5%)	6 (40.0%)	0.673	0.69	0.12	3.96
	No	5 (62.5%)	9 (60.0%)				
Attitude—Restrict priority antibiotics to human use only	No	4 (50.0%)	6 (40.0%)	0.645	1.50	0.27	8.45
	Yes	4 (50.0%)	9 (60.0%)				

**Table 6:** Proportion of operators of establishments keeping food-producing animals who correctly or incorrectly responded to knowledge, attitudes, and practices questions on AMR and AMU.

**Knowledge Factors**		**N (%)**
O_B1	Do you know who is authorized to issue a prescription?	159 (96.4)
O_B2	Do you know what antimicrobial medicines are?	147 (89.1)
O_B3	Do you know what antimicrobial residues are?	126 (76.4)
O_B4	Do you know what the withdrawal period for antimicrobial medicine is?	150 (90.0)
O_B5	Do you know what antimicrobial resistance is?	119 (72.1)
O_B6	Did you know that consuming food of animal origin before the withdrawn period can contribute to the development of antimicrobial resistance in humans?	116 (70.3)
O_B7	Can the development of antimicrobial resistance be reduced by avoiding the excessive use of antimicrobials in animal production?	128 (77.6)
O_B8	Are you aware of specific antimicrobials that target particular diseases?	119 (72.1)
O_B9	Do you think antimicrobials can transfer to humans through the consumption of animal products?	120 (72.7)
O_B10	Do you think antimicrobials are effective in treating both bacterial and viral infections?	29 (17.6)
O_B11	Do you think antimicrobials can have side effects?	124 (75.2)
O_B12	Do you think that all antimicrobials produce the same therapeutic effect on animal diseases?	111 (67.3)
O_B13	Do you think zoonotic agents in animals can develop resistance to antimicrobials?	114 (69.1)
O_B14	Do you think antimicrobial resistance in animals a significant concern for public health?	139 (84.0)
**Attitude Factors**		**N (%)**
O_C1	Do you think that antimicrobials used accidentally could contribute to antimicrobial resistance?	97 (58.8)
O_C2	Do you think insufficient dosage can contribute to antibiotic resistance?	101 (61.2)
O_C3	Do you think that limiting the use of antimicrobials could cause more harm than benefits?	54 (32.7)
O_C4	Do you think antimicrobials should be stored in restricted areas and accessed only by specific staff when needed?	15 (9.1)
O_C5	Do you reserve antimicrobials for later use?	150 (90.9)
O_C6	Do you use any alternatives to antibiotics in animal feed such as probiotic, organic acids, or others?	91 (55.2)
O_C7	Would you use fewer antimicrobials if you knew that accidental use could hinder recovery in the future?	141 (85.5)
**Practice Factors**		**N (%)**
O_D1	Do you have an agreement with a veterinarian to monitor your establishment?	120 (72.7)
O_D2	If you have answered “Yes” to question D1, does the veterinarian monitoring your establishment work for a veterinary pharmaceutical company?	97 (58.8)
O_D3	If you have answered “No” to question D1, do you receive advice from veterinarians working in veterinary pharmaceutical companies?	70 (42.4)
O_D4	Have you used antimicrobial medicines in your animals on your own initiative?	106 (64.2)
O_D5	Do you reserve antimicrobials for later use?	62 (37.6)
O_D6	Do you follow recommendations for the use of antimicrobials from non-veterinarians?	145 (87.9)
O_D7	Check the expiry date of antimicrobial medicines before purchasing them?	149 (90.3)
O_D8	Do you adhere to the antimicrobial withdrawal period?	160 (97.0)
O_D9	Do you increase the dose and frequency of antimicrobials when there are no signs of recovery?	128 (77.6)
O_D10	Do you stop administering antimicrobials when there is evidence of improved animal health?	59 (35.8)
O_D11	Are prescriptions issued in accordance with the instructions of the veterinarian who issued them?	159 (96.0)
O_D12	Is there a responsible person in your establishment to administer medicines?	142 (86.1)
O_D13	Do you apply biosecurity measures on your farm?	99 (60.0)
O_D14	If your answer to question D15 is “Yes”, what biosecurity measures do you apply to your establishment (including situations describing actions related to biosecurity)?	50 (30.3)

**Table 7:** Logistic regression analysis of attitude parameters associated with good practices (use of alternatives to antibiotics).

Variables	Adjusted Odds Ratio	95% Confidence Interval	
		Lower	Upper
Conclusion of an agreement with a veterinarian monitoring the establishment	2.56	1.22	5.56
Randomly used antimicrobials could contribute to antimicrobial resistance	0.95	0.47	1.96
Do you think insufficient dosage can contribute to antibiotic resistance	2.44	1.19	5.00
Do you think that limiting antimicrobials may cause more harm than benefits	2.33	1.12	4.76

**Table 8:** Logistic regression analysis of attitude parameters associated with good practices (implementation of biosecurity measures).

Variables	Adjusted Odds Ratio	95% Confidence Interval	
		Lower	Upper
Conclusion of an agreement with a veterinarian monitoring the holding	1.43	3.45	5.92
Randomly used antimicrobials could contribute to antimicrobial resistance	1.85	0.82	4.17
Do you think insufficient dosage can contribute to antibiotic resistance	2.33	1.03	5.26
Do you think that limiting antimicrobials may cause more harm than benefits	3.13	1.47	6.67

**Table 9:** Cross-tabulation between the variable “contract with a veterinarian who monitors the establishment” and other attitude variables using the chi-square test to estimate the level of significance (*p*-value) in each case.

		D1—Contract with a Veterinarian Who Monitors the Establishment		*p*-Value	Odds Ratio for (No/Yes)	95% Confidence Interval	
		No	Yes			Lower	Upper
Implementation of biosecurity measures	No	36 (80.0%)	79 (65.8%)	0.078	2.08	0.91	4.72
	Yes	9 (20.0%)	41 (34.2%)				
Attitude—Random AMU contributes to AMR	No	27 (60.0%)	41 (34.2%)	0.003	2.89	1.43	5.85
	Yes	18 (40.0%)	79 (65.8%)				
Attitude—Insufficient dosage can contribute to AMR	No	22 (48.9%)	42 (35.0%)	0.103	1.78	0.89	3.56
	Yes	23 (51.1%)	78 (65.5%)				
Attitude—Limiting antimicrobials may cause more harm than benefits	No	35 (77.8%)	76 (63.3%)	0.078	2.03	0.92	4.49
	Yes	10 (22.2%)	44 (36.7%)				
Use any alternatives to antibiotics in feed	No	29 (64.4%)	45 (37.5%)	0.002	3.02	1.48	6.17
	Yes	16 (35.6%)	75 (62.5%)				

**Table 10:** Association between socio-demographic characteristics of food-producing animal establishment operators with knowledge, attitudes, and practices using the Kruskal–Wallis test.

		Knowledge		Attitudes		Practices	
Socio-Demographics		Median (IQR)	*p*-Value	Median (IQR)	*p*-Value	Median (IQR)	*p*-Value
Age (years)	18–30	0.71 (0.29)	0.646	0.71 (0.43)	0.371	0.75 (0.31)	0.196
	31–45	0.79 (0.27)		0.57 (0.28)		0.66 (0.19)	
	46–60	0.79 (0.36)		0.57 (0.28)		0.69 (0.25)	
	>61	0.86 (0.29)		0.57 (0.28)		0.75 (0.18)	
Educational level	Elementary school graduate	0.61 (0.43)	0.025	0.43 (0.14)	0.032	0.75 (0.31)	0.328
	Middle school graduate	0.71 (0.36)		0.43 (0.28)		0.72 (0.12)	
	High school graduate	0.86 (0.29)		0.57 (0.28)		0.69 (0.18)	
	Highest education	0.79 (0.29)		0.57 (0.28)		0.69 (0.19)	
	Master’s degree and doctorate	0.93 (0.07)		0.71 (0.29)		0.75 (0.12)	
District where the establishment is located	Nicosia	0.79 (0.29)	0.010	0.57 (0.28)	0.008	0.75 (0.19)	0.135
	Larnaca	0.86 (0.22)		0.57 (0.14)		0.69 (0.20)	
	Limassol	0.75 (0.22)		0.43 (0.14)		0.75 (0.18)	
	Ammochostos	0.75 (0.45)		0.43 (0.14)		0.63 (0.21)	
	Paphos	0.71 (0.40)		0.57 (0.35)		0.63 (0.19)	
Target species	Bovine animals	0.86 (0.36)	0.000	0.57 (0.28)	0.000	0.69 (0.19)	0.057
	Caprine and ovine animals	0.71 (0.29)		0.57 (0.14)		0.69 (0.19)	
	Porcine animals	0.90 (0.14)		0.86 (0.26)		0.78 (0.24)	
	Poultry	0.86 (0.16)		0.71 (0.43)		0.72 (0.15)	
Professional experience (years)	<4	0.68 (0.45)	0.070	0.36 (0.39)	0.075	0.60 (0.30)	0.178
	5–8	0.71 (0.43)		0.43 (0.42)		0.75 (0.19)	
	9–12	0.79 (0.41)		0.57 (0.28)		0.69 (0.19)	
	>13	0.86 (0.29)		0.57 (0.28)		0.69 (0.18)	

## Data Availability

Data supporting reported results, including the questionnaire used (in Greek language), are available following communication with the study authors.
